# Anionic Lipids: A Pipeline Connecting Key Players of Plant Cell Division

**DOI:** 10.3389/fpls.2019.00419

**Published:** 2019-04-12

**Authors:** Marie-Cécile Caillaud

**Affiliations:** Laboratoire Reproduction et Développement des Plantes, Université de Lyon, ENS de Lyon, UCB Lyon 1, CNRS, INRA, Lyon, France

**Keywords:** cytokinesis, phosphoinositides, electrostatics, cell division, plant, division plane, anionic lipids

## Abstract

How cells position their division plane is a critical component of cell division. Indeed, it defines whether the two daughter cells divide symmetrically (with equal volumes) or not, and as such is critical for cell differentiation and lineage specification across eukaryotes. However, oriented cell divisions are of special significance for organisms with cell walls, such as plants, because their cells are embedded and cannot relocate. Correctly positioning the division plane is therefore of prevailing importance in plants, as it controls not only the occurrence of asymmetric cell division, but also tissue morphogenesis and organ integrity. While cytokinesis is executed in radically different manners in animals and plants, they both rely on the dynamic interplay between the cytoskeleton and membrane trafficking to precisely deliver molecular components to the future site of cell division. Recent research has shown that strict regulation of the levels and distribution of anionic lipids, which are minor components of the cell membrane’s lipids, is required for successful cytokinesis in non-plant organisms. This review focused on the recent evidence pointing to whether such signaling lipids have roles in plant cell division.

## Introduction

**FIGURE 1:**
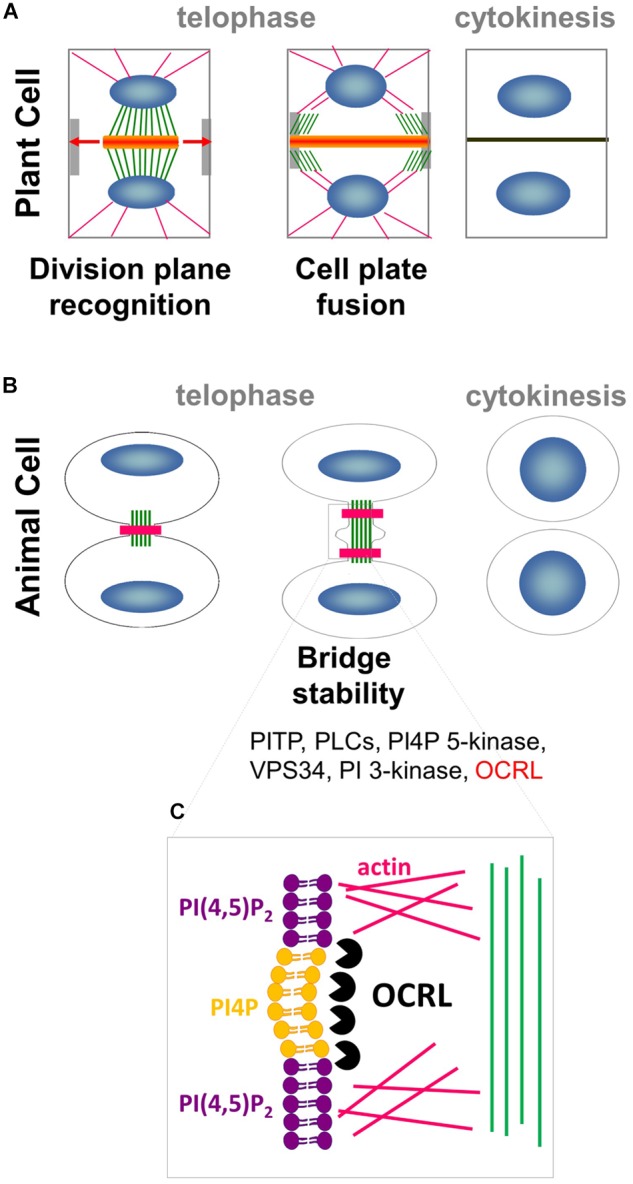
Interplay between PIPs, cytoskeleton and membrane trafficking. **(A)** In plant cell, phragmoplast of MTs (green) and actin filaments (pink) arises between daughter nuclei (blue), and guides the movement of Golgi-derived vesicles (orange) containing cell wall materials to the cell plate. As cytokinesis proceeds, the phragmoplast expands laterally (arrows) until it fuses with the parental PM and cell wall at the cortical division site (light gray). **(B)** In animal cells, a contractile actomyosin ring (pink) that mediates ingression of the cleavage furrow is formed. Successful cytokinesis is completed with abscission of the two cells at the midbody ring (green). Note that most of the PIPs modifiers (in black) are conserved in plant whereas OCRL (in red) has no homolog in the green lineage. **(C)** In animal cells, PI(4,5)P_2_ hydrolysis via OCRL (black packman) is important for normal cytokinesis abscission to locally remodel the F-actin cytoskeleton (pink) in the intercellular bridge during telophase.

Oriented cell divisions are significant steps in plant morphogenesis because plant cells are embedded in cell walls and cannot relocate. The establishment of the division plane, which involves the construction of the cell plate, is therefore essential in this process in plants. The cell plate is formed between the dividing cells by the directed delivery of vesicles along microtubules of the phragmoplast (Figure 1A). *De novo* microtubule formation at the outer border of the phragmoplast, together with depolymerization of microtubules at its center, allows for the centrifugal expansion and guidance of the cell plate toward the parental cell wall (Jurgens, 2005; Figure 1A).

Cytokinesis in metazoans is strikingly different from that in plants. In animal cells, components of the cleavage furrow are recruited to the cell equator, where microtubules then reorganize to form a region of bundles between the chromosomes termed the midzone (Glotzer, 2005; Eggert et al., 2006). A critical succeeding step is the formation of the midbody, a contractile actomyosin ring which mediates the ingression of the cleavage furrow (Figure 1B). Successful cytokinesis is completed with the abscission of the two cells at the midbody ring, which consists of overlapping, antiparallel bundles of microtubules (Eggert et al., 2006; Steigemann and Daniel, 2009). While cytokinesis is executed in radically different manners in animals and plants, they both rely on the dynamic interplay between the cytoskeleton and plasma membrane (PM) to precisely deliver the correct molecular components to the future site of cell division.

**FIGURE 2:**
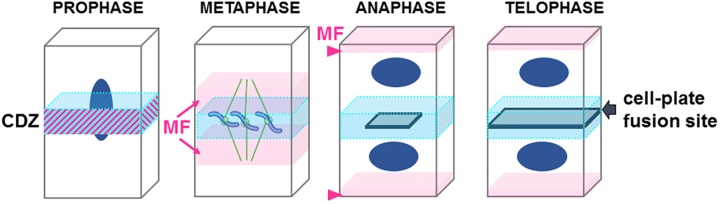
Cartoon representing the step of cell division in plant. Central division zone (CDZ) in blue, Actin microfilaments (MF) in pink, nucleus in dark blue, growing cell plate in gray. Note that in prophase actin MF localizes at the CDZ while it is excluded shortly before metaphase (ADZ) when actin “twin peaks” can be observed (pink arrows). In anaphase/metaphase transition a strong actin MF signal is observed at the apical/basal region of the cells (arrowhead). At the end of the telophase the expanding cell plate will join the PM of the mother cell by excluding proteins from the CDZ and therefore forming the cell-plate fusion site (gray arrow).

Before the initiation of mitosis, the future site of plant cell division is determined at the so-called “cortical division zone” (CDZ, Figure 2). Memory of this region is preserved throughout plant cell divisions, and its location in one dividing cell coincides with the site of its later division (Muller and Jurgens, 2016).

**FIGURE 3:**
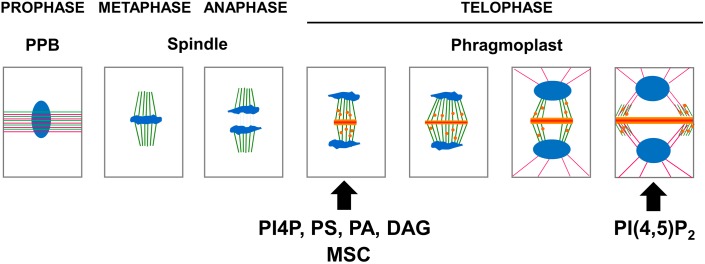
Schematic representation of the apparition of the anionic lipid at the cell plate. In preprophase, cortical microtubules (green) and actin filaments (pink) reorganize into a cortical ring positioned at the center of the cell named the preprophase band (PPB). At the end of prophase an acentriolar spindle is formed. After the separation of the chromosomes (blue), the transport along the phragmoplast of vesicle (orange) derived from the Golgi apparatus and, or *trans*-Golgi network (TGN) accumulate in the center of the cell, forming the cell plate. At this stage, PI4P, PS, PA, and DAG could be observed using their respective biosensors. The gradual expansion of the phragmoplast allows growth of the cell plate toward the periphery of the mother cell. When the phragmoplast reaches its maximum expansion, the neo-formed cell plate connects to the primary wall of the mother cell. At this stage, PI(4,5)P_2_ could be observed using its corresponding biosensor. The timing of apparition at the cell plate of each anionic lipids as well as membrane surface charge biosensor (MSC) are represented (arrow).

In preprophase, cortical microtubules and actin filaments reorganize into a cortical ring positioned at the center of the cell called the preprophase band (PPB, Figure 3). At the end of prophase, the nuclear envelope breaks down, which is then followed by the formation of an acentriolar spindle (Figure 3). The position of the spindles in both metaphase and anaphase seemingly responds to signals that maintain the division plane. The nature of these cues remains unknown, but they are expected to be produced in the CDZ. In telophase, the construction of a new intermediate wall between the two daughter cells involves a plant-specific structure (Figure 1A, 3), the phragmoplast (Smertenko et al., 2017). The phragmoplast is composed of two sets of microtubules of opposite polarity, actin filaments, and a “cell-plate assembly matrix” within which the cell plate is formed (Segui-Simarro et al., 2004). Vesicles derived from the Golgi apparatus and/or *trans*-Golgi network (TGN) are then transported along the microtubules of the phragmoplast and accumulate in the center of the cell, forming the cell plate (Muller and Jurgens, 2016). The gradual expansion of the phragmoplast permits the growth of the cell plate toward the periphery of the mother cell (Figure 1A, 2, 3). When the phragmoplast has expanded to its maximum extent, the newly formed cell plate connects to the primary wall of the mother cell, causing the splitting into two daughter cells (Smertenko et al., 2017). The cell plate fusion site bisects the CDZ during late cytokinesis via a yet-unknown mechanism (Figure 2). The distinct molecular composition of the CDZ versus that at the cell plate fusion site (Buschmann et al., 2006; Vanstraelen et al., 2006) suggests that the cell plate fusion site and CDZ represent distinct PM domains. The loss of membrane-bound actin filaments (Bo and Palevitz, 1992; Baluska et al., 1997) and the exclusion of some proteins from the CDZ (Vanstraelen et al., 2006) suggest that the formation of the CDZ causes local changes in the lipid composition of the PM.

It was recently found that membrane lipids are not just passive bystanders during cell division. Instead, during animal cytokinesis the lipid composition of the PM is remodeled, and the PM is enriched with several anionic lipids at the site of the cleavage furrow (Nezis et al., 2010). Anionic phospholipids are minor membrane lipids, but are critically important in signaling events. The main anionic phospholipids are phosphatidylinositol phosphates (PIPs, also known as phosphoinositides), phosphatidylserine (PS), and phosphatidic acid (PA). PIPs include seven members derived from the reversible phosphorylation or dephosphorylation of the inositol rings at the 3rd, 4th, and 5th positions of phospholipid molecules. Phosphatidylinositol 4-phosphate (PI4P) and phosphatidylinositol 4,5-bisphosphate [PI(4,5)P_2_] are two major phosphoinositides that function relatively independently as the determinant lipids of the plant cell’s PM (Simon et al., 2016). PIPs guide downstream signaling through the binding of proteins that harbor dedicated PIP-binding domains, or through electrostatic interactions. PIP patterning occurs through the tight regulation of the metabolic pathway of given PIPs that guides the sites of their production or degradation. Hydrolysis of PI(4,5)P_2_ by membrane-bound phospholipase C (PLC) produces diacylglycerol (DAG) and inositol trisphosphate. Although inositol trisphosphate diffuses into the cytosol, DAG remains within the PM due to its hydrophobic properties. Acidic phospholipids are precursors of the second messengers involved in key signaling cascades, or are second messengers themselves. They regulate the localization and activation of many proteins, and are involved in practically all membrane trafficking events. PA acts as a biosynthetic precursor for the formation of all acylglycerol lipids in the cell. The conversion of PA into DAG is the commitment step in the production of phosphatidylcholine (PC), phosphatidylethanolamine (PE), and PS.

In metazoans, local increases in PI(4,5)P_2_ levels modify the amounts of actin filaments gathered at the cell equator, which controls cytokinetic abscission (Dambournet et al., 2011). Local production of PI(4,5)P_2_ is controlled by HsOCRL 5-phosphatase, which converts PI(4,5)P_2_ into PI4P (Figure 1B,C; Dambournet et al., 2011; Echard, 2012). This finding suggests that localized PI4P/PI(4,5)P_2_ acts as a signaling hub in metazoans by promoting proper actin cytoskeleton organization in preparation for cell division, and thus directing membrane trafficking to the division plane (Logan and Mandato, 2006). A growing body of evidence points toward these signaling lipids having roles in plant cell division. This review focused on recent advances in our understanding of the roles of anionic lipids in plant cell division.

## Anionic Lipid Patterning During Plant Cell Division

The localization of anionic lipids has long been a tedious process to study because it relied mainly on immunolocalization experiments that often failed due to losses in preserved membrane integrity. An astonishing new view of the spatial and temporal patterning of anionic lipids emerged from advances in fluorescence microscopy. Genetically encoded biosensors consisting of fluorescent-tagged lipid-binding domains that specifically interact with a given anionic lipid were used to map the anionic lipid patterning inside plant cells. The use of native PIP-binding domains to report the localization of PIPs allowed the PIP patterning during cell division to be visualized in more physiologically accurate conditions *in vivo* (Figure 3).

### PI4P Is Present at the Cell Plate, While PI(4,5)P_2_ Is Excluded From It

A fluorescent-tagged version of the PH domain of the human protein PI4P adaptor protein-1 (FAPP1) was used to visualize the major pool of PI4P in plant cells (van Leeuwen et al., 2007; Simon et al., 2014; Tejos et al., 2014). PI4P localizes at the PM and at the TGN in interphasic root cells of *Arabidopsis* (Simon et al., 2014). In BY2 cells, the PI4P biosensor labeled the cell plate throughout cell division (Vermeer et al., 2009). Using a similar approach for the visualization of the PH domain of human PLCd1 (or the C-terminal domain of the TUBBY protein) fused to a fluorescent tag showed that PI(4,5)P2 strictly localizes at the PM, and is absent from the endosomal compartments in *Arabidopsis* root cells (Simon et al., 2014; Tejos et al., 2014). In dividing BY2 cells, PI(4,5)P_2_ is excluded from the growing cell plate (van Leeuwen et al., 2007). At the final stage of cytokinesis in BY2 cells, when the phragmoplast reaches the PM of the parental cell, YFP-PH^*PLCd1*^ accumulates on the leading edges of the cell plate (van Leeuwen et al., 2007).

This observation was recently confirmed using time-lapse imaging of the *Arabidopsis* root meristem (Doumane et al., 2017), taking advantage of a collection of biosensors expressed stably in *Arabidopsis* (Simon et al., 2014). While both PI4P and PI(4,5)P_2_ were present at the plant PM, only PI4P was localized at the center of the cell during anaphase (Figure 3; Simon et al., 2014). In contrast, PI(4,5)P_2_ was found to be excluded from the cell plate until late telophase, when the cell plate was joining to the mother cell’s wall (Figure 3; Simon et al., 2016). These results suggest that the cell plate becomes rich in PI(4,5)P_2_ just prior to undergoing total fusion with the mother cell’s PM (van Leeuwen et al., 2007; Simon et al., 2016), which points toward PI(4,5)P_2_ having a role in PM identity. Indeed, PI(4,5)P_2_, which is only detectable at the PM, regulates endocytosis and exocytosis. The absence of PI(4,5)P_2_ prior to the full attachment of the cell plate to the mother cell’s PM could be a key signaling component during the cell plate maturation process, evolving from “TGN structures” toward a functional PM. It was recently shown that the polarity of the auxin efflux carrier PIN2 was re-established via localized clathrin-mediated endocytosis after cytokinesis was completed (Glanc et al., 2018). Investigating the role of anionic lipids in such processes should help us to better understand the membrane lipid environment necessary for such plant-specific phenomena.

Membrane surface charges in the root epidermis of *Arabidopsis* were also mapped to assess the importance of membrane electrostatics in plants (Simon et al., 2016; Platre and Jaillais, 2017). Using membrane surface charge biosensors (MSC), the PM was shown to have a overall positive charge, a unique and intrinsic property of this membrane that contributes to its identity (Simon et al., 2016). Simultaneous imaging *in vivo* of dividing root cells in *Arabidopsis* showed that while the membrane surface charge probe was recruited along with the PI(4)P biosensor to the cell plate, the PI(4,5)P_2_ biosensor was excluded from it (Simon et al., 2016). This result suggests that PI(4,5)P_2_ helps in the establishment of a highly charged electrostatic field, at least at the cell plate. In contrast, PI(4)P accumulation is correlated with high membrane electrostatic charges at the PM and cell plate, suggesting that it could be important in establishing membrane surface charges (Simon et al., 2016).

A controversial polarity of both PI4P and PI(4,5)P_2_ in the PM to the apical/ basal sides of root epidermal cells was recently reported (Tejos et al., 2014). This bipolar localization pattern was displayed particularly strongly in cells expressing only low levels of reporters, in which the perturbation of cellular function by the expression of reporters was minimal (Tejos et al., 2014). However, the determination of the “polarity indices” in *Arabidopsis* root tip cells suggested that the localization of PIPs reporters do not differ between lateral and apical/basal sides of the cell compared with non-polar controls (Simon et al., 2016). The assumption that confocal images of root cells might have been biased because of the topology of these cells was therefore favored over the actual occurrence of these bipolar localization patterns (Simon et al., 2016). However, based on imaging of dividing *Arabidopsis* cells *in vivo* (Simon et al., 2016), the possibility that the level of PI(4,5)P_2_ localized at the apical/basal region of the dividing cells might vary during cell division processes cannot be excluded. Clear quantification of this phenomenon needs to be performed to conclude whether or not PI(4,5)P_2_ polarization really occurs, in particular in the context of plant cell division.

### PA Localizes at the PM and Is Present at the Cell Plate

Using the PA-binding domain of yeast SNARE Spo20p fused to fluorescent proteins, it was previously shown in tobacco pollen tubes that PA accumulates in the subapical region of the cytosolic leaflet of the PM (Potocky et al., 2014). Transgenic *Arabidopsis* lines expressing fluorescent-tagged variants of the recently developed “PA biosensor with superior sensitivity” (PASS) (Zhang et al., 2014) were next generated (Platre et al., 2018b). Using this approach, it was shown that PA also localize at the PM in *Arabidopsis* sporophytic tissues in root and shoot (Platre et al., 2018b). In dividing *Arabidopsis* cells, PA localizes at the cell plate early on in its formation, where it colocalizes with the endocytic dye FM4-64 (Figure 3; Platre et al., 2018b).

### PS Localizes at the PM and Along the Endocytic Pathway, and Is Present at the Cell Plate

In *Arabidopsis*, the PH domain of human EVECTIN2 (PH^*EVCT2*^) and the stereospecific PS-binding C2 domain of bovine lactadherin (C2^*LACT*^) were used to visualize PS inside the plant cell (Platre et al., 2018b). Using these biosensors, PS was shown to localize at the PM and along the endocytic pathway (Platre et al., 2018b). Using a root-tracking system (Doumane et al., 2017), the PS biosensor mCIT-C2^*LACT*^ was localized at the forming cell plate during cytokinesis (Figure 3) together with PA and PI4P, therefore correlating with the acquisition of electrostatic identity by the cell plate (Platre et al., 2018b).

### DAG, a Derivative of Phosphoinositide Metabolism, Is Present at the Cell Plate

The biosensor for DAG consists of a fusion between the cysteine-rich 1a domain of human PKCg (C1^*aPKC*^) that specifically binds to DAG and the yellow fluorescent protein (YFP) (Vermeer et al., 2017). In stable *Arabidopsis* lines, YFP–C1^*aPKC*^ fluorescence was found at the TGN, as was also observed in tobacco BY-2 cells expressing the DAG biosensor (Vermeer et al., 2017). In epidermal root cells, YFP–C1^*aPKC*^ labeled the PM. Interestingly, the PM localization of the biosensor progressively vanished as the epidermal cells started to elongate (Vermeer et al., 2017), as was also observed for the PS biosensors (Platre et al., 2018a). Following cell divisions in BY2 cells using the YFP–C1^*aPKC*^ biosensor, DAG was found to label the newly formed cell plate right up until fusion with the parental PM occurred (Figure 3; Vermeer et al., 2017). Colocalization with the endosomal dye FM4-64 suggested that YFP–C1^*aPKC*^ appeared at the newly formed cell plate only slightly later, as observed for PI4P biosensors (van Leeuwen et al., 2007; Vermeer et al., 2009).

### PI3P Is Present in the Late Endosome, but Is Excluded From the Cell Plate

The localization of PI3P was assessed using a fluorescent-tagged FYVE domain (Vermeer et al., 2006). In interphasic root cells of *Arabidopsis*, PI3P is localized in the late endosome in the multi-vesicular body (MVB) compartment (Simon et al., 2014). During cell division in BY2 cells, clouds of PI3P-enriched vesicles were visible as a belt surrounding the newly formed cell plate, but these PIP3-enriched vesicles were absent from the vicinity of the growing cell plate (Vermeer et al., 2006). This result suggested that PI3P is involved in the vesicle trafficking to/from the newly formed cell plate, but is itself excluded from it.

### Localization of PI(3,5)P2 in Dividing Cells?

Using a tandem repeat of the cytosolic phosphoinositide-interacting domain (ML1N) of the mammalian lysosomal transient receptor potential cation channel Mucolipin 1 fused to a fluorescent tag (tagRFP-2xML1N), PI(3,5)P_2_ was shown to be mainly localized on late endosomes, but was also sometime observed at the tonoplast or the TGN of root cells in stable lines of *Arabidopsis* (Hirano et al., 2017). While additional biosensors were recently developed to visualize PI(3,5)P_2_ in *Arabidopsis* (Hirano et al., 2018), none of them have yet been used to determine the subcellular localization of this low-abundance anionic lipid in the context of cell division.

## PIP Metabolism During Cell Division

**FIGURE 4:**
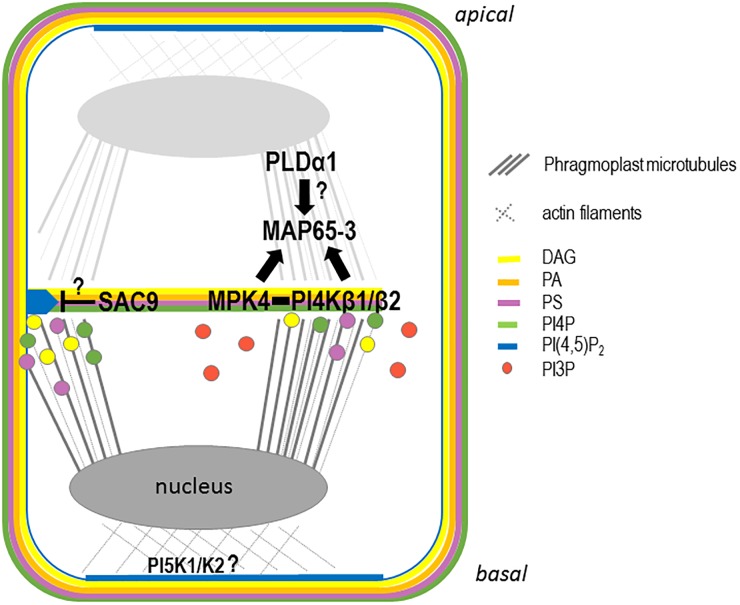
Schematic representation of the anionic lipid metabolism during cell plate attachment. In late telophase, PI4P, PA, PS, and DAG are present at the growing cell plate probably through trafficking from the golgi and, or TGN. Additional synthesis of PI4P directly at the cell plate could be achieve via the PI4PKβ1/β2 enzymes. At this stage, PI(4,5)P_2_ is accumulating at the apical/basal region of the cell via the action of the PIP5K1 and PIP5K2 enzymes, leading to an accumulation of the actin cytoskeleton at the apical/basal region of the cell. In parallel, PI4PKβ1/β2 produce locally PI4P at the growing cell plate regulating MAP65-3 directly or indirectly via MPK4. At this stage, PLD1 might regulates the PA pattering at the cell plate also impacting the function of MAP65 family members. During cell plate growth, PI(4,5)P_2_ is excluded from the cell plate. When the cell plate attached to the mother cell wall, the PI(4,5)P_2_ diffusion to the entire cell plate might be restricted by the action of a 5-phosphatase such as SAC9.

To understand whether the aforementioned anionic lipids are produced at the growing cell plate or if their presence there reflects the results of targeted trafficking of vesicles enriched in these anionic lipids (i.e., via the TGN) to the growing cell plate, the enzymes that produce these anionic lipids have to be localized in the context of plant cell division. Recent advances in our understanding of PIP metabolism highlight its role in determining the specific anionic lipid patterning observed during cell division (Figure 4).

### PI4P Metabolism Regulates Phragmoplast Expansion

Double mutants in the redundant PI-kinases PI4Kβ1 and PI4Kβ2 displayed cytokinetic defects, characterized by vesicles not fusing to the nascent cell plate and being present close to aborted cell wall stubs (Kang et al., 2011). In these double mutants, the trafficking of the plant specific syntaxin KNOLLE at the cell plate is impaired, and the phragmoplast microtubules that guide membrane trafficking at the cell plate are over-stabilized (Lin et al., 2019). PI4P formation by PI4Kβ1 is required for the correct localization of the MICROTUBULE ASSOCIATED PROTEIN 65-3 (MAP65-3) at the leading edge of the expanding cell plate (Lin et al., 2019). The functional mCherry-PI4Kβ1 fusion protein decorates the cell plate early in its formation and becomes concentrated at the growing edges of the cell plate during its expansion (Lin et al., 2019). Based on the physical interaction between PI4Kβ1 and the cytokinesis regulator MAP kinase-4 (MPK4), and on similar cytokinetic defects observed in both pi4kβ1/pi4kβ2 and mpk4 mutants, it was proposed that PI4Kβ and MPK4 act synergistically to control phragmoplast dynamics (Lin et al., 2019).

### PI(4,5)P_2_ Metabolism Is Tightly Regulated During Cell Division

PIP5K1 and PIP5K2 are the most active PI4P 5-kinases among the ones ubiquitously expressed (Stenzel et al., 2008), and are likely making a major contribution to the production of PI(4,5)P_2_ (Tejos et al., 2014). Recent evidence leads toward a roles of these PI4P-5-kinases in asymmetric cell division, in particular during embryogenesis (Tejos et al., 2014). Aberrant cell division orientation as early as the two-celled embryo stage in the *pi5k1*/*pi5k2* double mutant results in embryos with an inaccurate apical/basal axis and severe patterning defects (Tejos et al., 2014). Functional translational fusions between the PIP5K1 or PIP5K2 and the YFP were next generated, to assess their subcellular localizations (Ischebeck et al., 2013). The expression patterns of the fusion proteins under the control of their respective native promoters in the root apical meristem (Tejos et al., 2014) resembled those previously found using the transcriptional reporters (Elge et al., 2001). It is tempting to conclude from previously published studies that YFP-PIP5K1 and YFP-PIP5K2 are enriched in the apical–basal localized PM, in particular in dividing cells and at the corresponding fully expanded cell plate (Figure 4; Tejos et al., 2014). However, their dynamic localization through time and space during cell division still needs to be studied to determine whether these two kinases are indeed the ones responsible for the localized increase in PI(4,5)P_2_ at the newly formed cell plate after it becomes completely attached to the mother cell’s PM.

Phosphatidylinositol 4,5-bisphosphate accumulates transiently at the cell plate, more precisely at its leading edges prior to total fusion with the mother cell’s PM (van Leeuwen et al., 2007; Simon et al., 2016). The local decrease in the PI(4,5)P_2_ concentration in the maturation zone of the expanding cell plate is therefore necessary to maintain cell plate integrity. The local hydrolysis of PI(4,5)P_2_ could be achieved via different enzymatic pathways. Among them, SAC9, a 5-phosphatase that belongs to the SUPPRESSOR OF ACTIN (SAC) family (Williams et al., 2005), stands out as a good candidate for being involved in the local control of PI(4,5)P_2_ during cell plate extension. The cellular ultrastructure of *sac9-1* mutants revealed extreme abnormalities in their cell wall and disproportionate membrane material in root cells (Vollmer et al., 2011). Abundant vesicles were associated with these irregular wall structures, which resembled cell wall stubs often observed for mutant impaired in cytokinesis process (Vollmer et al., 2011). The subcellular localization of SAC9 needs to be determined to conclude whether it is indeed the PI(4,5)P_2_ 5-phosphatase responsible for the restricted PI(4,5)P_2_ distribution during cell plate expansion (Figure 4).

The *phosphoinositide phospholipase C* gene family also participates in PI(4,5)P_2_ metabolism. While nine members of this family are present in *Arabidopsis* (named *PLC1* to *PLC9*), among these PLC2 was found to be particularly important for female gametophyte development and early embryogenesis (Di Fino et al., 2017). Allelic hemizygous *plc2-2* mutant plants displayed reduced seed number and embryos arrested at the pre-globular stage characterized by abnormal patterns of cell division (Di Fino et al., 2017). Although the activity of this enzyme toward PI(4,5)P_2_ has not yet been studied, it is worth mentioning that the precursor for PI(4,5)P_2_, PI4P can be a substrate for PLC to generate DAG (Zhang et al., 2013). This result agrees with the observed accumulation of DAG at the cell plate (Vermeer et al., 2017). Phenotypical analyses and subcellular localization studies of PLC and other enzymes involved in the metabolism of DAG would be useful to understand the roles of PI4P and PI(4,5)P_2_ during cell division.

### Phosphatidic Acid Metabolism Regulates Asymmetric Cell Division in *Zea mays*

Phosphatidic acid is produced by diacylglycerol kinase (DGK) through the phosphorylation of DAG or by phospholipase D (PLD), which hydrolyzes PE and PC into PA. The complexity of PA production and composition is revealed by the presence of seven DGK and 12 PLD isoforms in the *Arabidopsis* genome (Yao and Xue, 2018). While the role of PA metabolism in symmetric cell division has yet to be investigated, the contribution of the PLC and PLD pathways in asymmetric cell divisions was investigated using *Zea mays* as a model (Apostolakos et al., 2008). In maize, the stomatal complex comprises a pair of guard cells associated by a pair of “subsidiary cells,” which help regulate stomatal aperture size. The guard mother cell triggers the asymmetric division of neighboring subsidiary mother cells, with the smaller subsequent daughter cells being positioned next to the guard mother cell (Panteris et al., 2006).

Pharmacological tools were used to assess the contribution of PA metabolism in this model system. The use of butanol-1, a preferred substrate for PLD that leads to the transformation of the phosphatidyl into a primary alcohol rather than water and produces a phosphatidylalcohol instead of PA, provided a quantitative measurement of PLD activity *in vivo* (Munnik et al., 1995). Neomycin, an aminoglycoside antibiotic that binds with high affinity to several PIPs, including the main substrate for PLC, prevents the cleavage of PI(4,5)P_2_ by PLC (Gabev et al., 1989). The PLC inhibitor U73122 has been used in a number of studies of calcium signaling mechanisms and other PLC-dependent processes. However, this inhibitor also causes major collateral effects, such as the rapid inhibition of cytoplasmic streaming (den Hartog et al., 2001), which might directly affect the interpretation of the results. Using these combined pharmacological approaches, it was proposed that the PLC and PLD signaling pathways might be involved in the transduction and/or perception of the inductive stimulus delivered from the guard mother cells that induces the polar actin filament organization and asymmetric division of subsidiary mother cells (Apostolakos et al., 2008). In contrast, division plane determination in subsidiary mother cells did not appear to depend on PLC and PLD signaling pathways using these pharmacological approaches (Apostolakos et al., 2008).

In *Arabidopsis* suspension cells, actin and β-tubulin interact with GFP-PLDδ in pull-down experiments, suggesting that PLDδ acts as a hub between the microtubule and actin cytoskeletons in plant cells (Ho et al., 2009). In tobacco pollen, actin interacts and regulates PLDβ1 activity (Pleskot et al., 2010). Thus, PLD directly connects microtubules and actin cytoskeletons and PA regulates microtubules and actin through PA-binding proteins. Under abiotic stress, such as salt stress, PA links the PM and microtubules via MAP65-1 (Zhang et al., 2012). Activation of PLDα1 leads to the production of PA, which binds to MAP65-1, which is responsible for the bundling of microtubules (Zhang et al., 2012). The salt-sensitive phenotype observed in *pldα1* mutants was rescued by exogenous application of PA, but not in those of *map65-1*. This result showed that the interaction between PA and MAP65-1 is crucial for the organization of cortical microtubule in response to salt stress (Zhang et al., 2012). The authors propose that specific PLD enzymes and their product, PA, anchor the microtubule cytoskeleton to specific sites on the PM in response to stimulus leading to the rapid microtubule cytoskeleton rearrangement. The MAP65 family is composed of nine members in *Arabidopsis*, and two of these members, MAP65-3 and MAP65-4, play major roles in the cytokinesis process (Muller et al., 2004; Caillaud et al., 2008; Li et al., 2017). It would therefore be interesting to investigate whether, in addition to MAP65-1, other members of this family such as MAP65-3 and MAP65-4, are required for proper PA patterning in the expansion of the microtubule phragmoplast during cell division (Figure 4).

### Implication of the PI3P and PI(3,5)P_2_ Metabolism During Cell Division

Whereas most genomes, from humans to yeast, contain a single copy of the gene for the kinase producing PI(3,5)P_2_ (Odorizzi et al., 2000), the *Arabidopsis* genome codes for four FORMATION OF APLOID AND BINUCLEATE CELLS 1 genes (*FAB1A–D*), of which only *FAB1A* and *FAB1B* have a FYVE domain (Mueller-Roeber and Pical, 2002). Double mutants for *fab1a*/*fab1b* were describe to have a phenotype with male gametophyte lethality in *Arabidopsis* (Whitley et al., 2009; Serrazina et al., 2014). The role of the PI(3,5)P_2_ produced by FAB1 seems, however, to be minor, since the cell division defects observed in *fab1a*/*fab1b* double mutants are secondary to their vacuole morphology defects (Whitley et al., 2009).

Mutations in the PI(3,5)P_2_ phosphatase *FRAGILE FIBER 7*/*SAC1* that dephosphorylates PI(3,5)P_2_ into PI3P in plants lead to abnormal cell files in the piths of inflorescence stems in *Arabidopsis* (Zhong et al., 2005). The normally linear arrangements of these cell files are determined during the division of pith precursor cells (Zhong et al., 2005). Since the *fra7/sac1* mutation alters actin organization, it was suggested that the aberrant cell files observed in the *fra7/sac1* pith were triggered by abnormal F-actin organization during cell division (Zhong et al., 2005). While the localization of FRA7/SAC1 in dividing cells in unknown, FRA7/SAC1 is associated with the Golgi when it was transiently expressed in onion epidermal cells (Zhong et al., 2005). FRA7/SAC1 might therefore play a role in regulating the PI(3,5)P_2_ content of vesicles trafficked toward (or away from) the expanding cell plate. Four other SAC1-like were described to be orthologous to the yeast Fig4p and to be involved in PI(3,5)P_2_ metabolism at the tonoplast (Novakova et al., 2014). Functional analysis of SAC2-SAC5 proteins in the context of plant cell division would be key toward our understanding on the role of PI(3,5)P_2_ metabolism in this process.

Even though PI3P is not directly localized at the growing cell plate or at the PM, it was reported that Wortmannin, a non-specific, covalent inhibitor of PI 3-kinases, inhibits cell plate growth (Dhonukshe et al., 2006). In that previous study (Zhong et al., 2005), Wortmannin was used at a 10 μM concentration that might also have had an impact on the activity of PI-4 kinases (Simon et al., 2016). Thus, the role of PI3P during cell division has yet to be experimentally addressed.

### Lipid Transfer Proteins: The Case of the SEC14-Type PATELLIN (PATL) Proteins

SEC14 lipid transfer proteins are main regulators of phospholipid metabolism. Studies in yeast suggested that SEC14 lipid transfer proteins might act as cofactors for PIP-kinases, by helping them to recognize membrane-embedded substrates. Genomes of higher plants encode a great number of SEC14 domain containing proteins, often found in combination with other protein domains. In *Arabidopsis* root cells and tobacco BY-2 cells, the SEC14-type protein PATL1 localizes at the center of the cell plate—where maturation occurs—and binds to PIPs *in vitro*, suggesting a potential link between PIPs and membrane recycling during cell plate maturation (Peterman et al., 2004; Peterman et al., 2006). Another member of this protein family, PATL2 localization is also focused at the cell division plane in *Arabidopsis* along with MPK4, and has binding affinity for PIPs (Suzuki et al., 2016). This binding affinity is dependent on the phosphorylation of PATL2 by MPK4, suggesting a key role for the MAPK cascade during cytokinesis. In particular, PATL2/MPK4 might influence the membrane turnover during cell plate formation (Suzuki et al., 2016). PATL3, PATL4, and PATL6 are expressed and localized in dividing tissues, and PATL3p:GFP-PATL3 accumulates at newly fused cell plate at the end of cytokinesis (Tejos et al., 2018). However, no developmental abnormalities were detected in *patl3* single-mutants, indicating a manifest functional redundancy among the members of the *PATL* gene family (Tejos et al., 2018). When a *patl3* mutation (or artificial microRNAs targeting *PATL3*) was introduced into the quadruple *patl2456^−/−^* mutant, the resultant quintuple mutation was partially lethal. Phenotypes with stronger apical/basal embryo patterning were also observed in the *patl23456^−/−^* quintuple mutant, suggest a critical and redundant function for PATLs in organogenesis (Tejos et al., 2018). The *Arabidopsis* genome encodes about 30 SEC14 domain-containing proteins, most of which are uncharacterized, but which undoubtedly also play a role in the regulation of the PIP-patterning. The further study of lipid-interacting protein families should help us to better understand the mechanisms of membrane recycling and dynamics during cytokinesis, in particular during cell plate maturation.

## Regulation of Protein Localization During Cell Division

### Bridging of Actin and the PM During Plant Cell Division

In plant cells, F-actin dynamically re-localize as cell division progresses. F-Actin first concentrate at the CDZ together with microtubules to form the PPB in the early stages of mitosis, and then become excluded from it in late prophase to form an “actin-depleted zone” (Smith, 1999). In some cell types, either or both sides of the CDZ remain enriched with actin filaments, which forms the “actin twin peaks” (Smertenko et al., 2010). In cytokinesis in animals, a local increase in the level of PI(4,5)P_2_ modifies the amount of F-actin at the cell equator and controls abscission (Dambournet et al., 2011). Actin nucleators, such as ARP2/3, were shown to interact strongly with cell membranes in *Arabidopsis* (Kotchoni et al., 2009), and both profilin and ADF/cofilin can bind to PI(4,5)P_2_ in maize (Gungabissoon et al., 1998). Furthermore, PROFILIN1 from *Zea mays* (ZmPRO1) inhibits the hydrolysis of membrane PI(4,5)P_2_ by PLC (Staiger et al., 1993). In *Phaseolus vulgaris*, profilin interacts directly to PI-3kinases, suggesting that profilin may participate in membrane trafficking, and may also act to link the endocytic pathway and the reorganization of the actin cytoskeleton (Aparicio-Fabre et al., 2006).

While the role of such actin regulators is not yet known in the context of plant cell division, functional analysis of the plant formin 5 (AtFH5) revealed its role in the control of cytokinesis (Ingouff et al., 2005). A constitutively expressed AtFH5 fused to GFP was localized at the zone of cell division in *Arabidopsis* root cells. In particular, AtFH5 strongly localized at the expanding cell plate and gradually disappeared after the cell plate fused with the mother cell wall (Ingouff et al., 2005). Further, a mutation in *Atfh5* slowed down cell plate formation (Ingouff et al., 2005). There are 21 formin homologs in *Arabidopsis*, which can be divided into two clades (class I and II) (Deeks et al., 2002). Members of class II have an N-terminal domain with high sequence similarity to the phosphatase and tensin (PTEN) domain, which was found to facilitate lipid binding (Cvrckova et al., 2004). In *Physcomitrella patens*, class II formin 2A (For2A) is indeed targeted to the cell cortex by binding PI(3,5)P_2_ through its PTEN domain (van Gisbergen et al., 2012). Binding to PI(3,5)P_2_ was found to be required for targeting For2 to the PM, where it is localized to peculiar cortical structures (van Gisbergen et al., 2012). During plant cell division, For2A localizes at the leading edges of the phragmoplast and is enriched along F-actin that bridge the phragmoplast midzone with the cell cortex. This particular localization provides roadways for translocation of the myosin VIII-dependent to the peripheral microtubules (Wu and Bezanilla, 2014). As the phragmoplast expands centrifugally, peripheral microtubules intersect the F-actin that extent the distance between the leading edges of the phragmoplast and the cell cortex (Wu and Bezanilla, 2014). This result suggests that microtubules may interact with F-actin connecting the cell cortex and the phragmoplast. Since PI(4,5)P_2_ also accumulates specifically at the cell plate at this stage, it is tempting to hypothesize that PIP composition mediates actin cytoskeleton polymerization at the edge of the growing cell plate to allow its proper fusion with the parental cell.

### Do PIPs Regulate the Protein Composition at the CDZ?

Recently, two of putative Rho-of-plants (ROP) GTPase-activating proteins (GAPs, ROP-GAPs) with putative PIP-binding domains (PHGAPs) were characterized for their interactions with POK1 (Stockle et al., 2016). GAPs rendering the GTPase inactive by stimulating the inherent GTPase activity of their respective small GTPase targets. The Rho-based regulatory mechanism controls several cellular processes in plant by establishing distinct subcellular domains important for the reorganization of the cytoskeleton, polarity and vesicle trafficking. In prophase, PHGAP1 and PHGAP2 fused to a fluorescent protein were found to localize uniformly at the PM (Stockle et al., 2016). From the meta-/anaphase transition until the completion of cytokinesis, PHGAP1 and PHGAP2 were observed to be enriched at the CDZ (Stockle et al., 2016). Loss of PHGAP function leads to an imprecise CDZ delineation characterized by tilted PPBs, which consequently affects POK1 positioning (Stockle et al., 2016). Since PHGAP1/PHGAP2 still need POK1 activity for their proper localization at the CDZ during mitosis (Stockle et al., 2016), PHGAPs and POK1 are proposed to act in a feedback loop, where PHGAPs act upstream of division plane selection and succeeding POK1 localization at the CDZ. The presence of a PIP-binding domain in the sequences of both PHGAP1 and PHGAP2 still needs to be analyzed to understand whether these proteins require interactions with specific PIPs at the CDZ for their localization and function.

## Conclusion

The concept of cell division orientation emerging from recent research is currently converging into the importance of the formation, at the cell equator, of membrane zonation that will guide the formation of the division plane. However, the intrinsic molecular mechanism by which such a zonation in the cell is formed remains unknown. The presence of negatively charged lipids at the PM and at the growing cell plate might regulate some of the key players in plant cell division. The coordination of cell division orientation might therefore involve the spatial and temporal regulation of PIP pattering for the recruitment, and thereby the function, of proteins in specific zones of the PM throughout plant cell division. To date, the study of the timing and distribution of protein localization throughout cell division has relied mainly on fragmented results often obtained from static observations of their localization. It is now technically possible to investigate what the spatial and, or temporal links are between cell division markers and the evolution of the actin and, or microtubule cytoskeletal dynamics, as well as the anionic lipid composition, during mitosis at a high resolution. As electron microscopy did in the past, super-resolution microscopy will be a key player in future research to help us understand highly dynamic polarized cells, such as dividing cells.

To complement the functional analysis of plants with a mutation in a given PIP metabolic enzyme, the impact of the global perturbation of anionic lipid patterning on the orientation of cell division can now be achieved. Recently developed systems in which the catalytic domain of a well-defined PIP phosphatase or kinase is targeted to the PM could allow the membrane lipid composition to be modified *in planta* (Rodriguez-Villalon et al., 2015; Simon et al., 2016). Using inducible systems, this approach will allow the importance of PIP patterning to the orientation of the cell division plane to be tested.

In a non-biased approach, it will be really important to isolate the endogenous plant proteins that are regulated by PIPs and/or MSC during plant cell division. Although technically challenging mass spectrometry discovery of PIP-interacting proteins during cytokinesis would have the potential to uncover unanticipated events that simply cannot be proposed *a priori* through a “classical” hypothesis-driven approach and might open new scientific horizons in yet unpredictable ways.

## Author Contributions

The author confirms being the sole contributor of this work and has approved it for publication.

## Conflict of Interest Statement

The author declares that the research was conducted in the absence of any commercial or financial relationships that could be construed as a potential conflict of interest.
